# The limits of metabolic heredity in protocells

**DOI:** 10.1098/rspb.2022.1469

**Published:** 2022-11-09

**Authors:** Raquel Nunes Palmeira, Marco Colnaghi, Stuart A. Harrison, Andrew Pomiankowski, Nick Lane

**Affiliations:** ^1^ Department of Computer Science, Engineering Building, Malet Place, University College London, WC1E 7JG, UK; ^2^ Department of Genetics, Evolution and Environment, University College London, Gower Street, London WC1E 6BT, UK

**Keywords:** origin of life, protocells, protometabolism, nucleotide cofactors, autocatalysis, mathematical model

## Abstract

The universal core of metabolism could have emerged from thermodynamically favoured prebiotic pathways at the origin of life. Starting with H_2_ and CO_2_, the synthesis of amino acids and mixed fatty acids, which self-assemble into protocells, is favoured under warm anoxic conditions. Here, we address whether it is possible for protocells to evolve greater metabolic complexity, through positive feedbacks involving nucleotide catalysis. Using mathematical simulations to model metabolic heredity in protocells, based on branch points in protometabolic flux, we show that nucleotide catalysis can indeed promote protocell growth. This outcome only occurs when nucleotides directly catalyse CO_2_ fixation. Strong nucleotide catalysis of other pathways (e.g. fatty acids and amino acids) generally unbalances metabolism and slows down protocell growth, and when there is competition between catalytic functions cell growth collapses. Autocatalysis of nucleotide synthesis can promote growth but only if nucleotides also catalyse CO_2_ fixation; autocatalysis alone leads to the accumulation of nucleotides at the expense of CO_2_ fixation and protocell growth rate. Our findings offer a new framework for the emergence of greater metabolic complexity, in which nucleotides catalyse broad-spectrum processes such as CO_2_ fixation, hydrogenation and phosphorylation important to the emergence of genetic heredity at the origin of life.

## 1. Introduction

The problem of whether genes or metabolism arose first has a long and contested history [1–7]. The question has limited value because nucleotide synthesis and polymerization necessarily require some form of protometabolism. A more useful approach is to investigate the protometabolic context in which genetic heredity first arose. One hypothesis for the origins of genetic heredity considers the emergence of RNA in protocells [8–12], where ‘autotrophic’ growth is driven by the continuous conversion of gases such as H_2_ and CO_2_ into organic matter via protometabolic pathways that prefigure the universal biosynthetic pathways [9]. These are assumed to exist in the absence of genes or enzymes, meaning that the chemistry of life is thermodynamically and kinetically favoured [13–15]. Experimental work now links geochemical CO_2_ fixation with the universal core of metabolism in bacteria and archaea [16–18]. Strikingly, core metabolic pathways including the acetyl CoA pathway [18–21], large parts of the Krebs cycle [16,22–24], glycolysis [25] and gluconeogenesis [26], the pentose phosphate pathway [17,25] and some amino acid biosynthetic pathways [22,27] do occur spontaneously without enzymes. Fatty acids [28–31] and critical sugars such as ribose [32,33] have also been synthesized non-enzymatically under relevant prebiotic conditions. The idea that metabolism emerged from a geochemical protometabolism therefore looks increasingly persuasive [34–37].

The advantage of this hypothesis is that it readily explains how various components that makeup protocells could come together to form a living system: the chemistry is driven *in situ* by a geologically sustained disequilibrium, primarily between H_2_ and CO_2_ [34,38–41]. But the idea that long, non-coded protometabolic pathways could arise spontaneously and be reliable enough to foster the emergence of the genetic code seems to strain credibility. In particular, nucleotide synthesis is missing from the pathways listed above. Purine nucleotide synthesis requires 12 steps, albeit with some repetitive chemistry, beginning with amino acids, sugar phosphates and an energy currency. There have only been limited experimental attempts to synthesize nucleotides prebiotically following these biological pathways [9,42,43]. Yet even if they are successful, could protometabolism really generate enough nucleotides to form RNA? If not, even the best-case scenario falls short of the requirements for the emergence of genetic heredity in autotrophic protocells. Using life as a guide to its own origin is then either misguided, or it must be possible for protocells to get better at making nucleotides before the emergence of genetic heredity.

The simplest resolution to the nucleotide-amplification problem is that nucleotides could catalyse their own synthesis, either directly or indirectly. Nucleotide-derived cofactors include coenzyme A, NADH, FADH, ATP, and the pterins and folates involved in CO_2_ fixation [44–46]. ‘Naked’ cofactors frequently catalyse the same chemistry as the holoenzymes, albeit more slowly [47–49]. It is therefore possible to imagine that nucleotides could form at trace levels within protocells and favour protocell growth and replication through simple catalysis. But could such catalysis increase nucleotide concentrations as well as protocell growth? If so, would the amplification of nucleotide synthesis in protocells depend on autocatalysis (nucleotide catalysis of nucleotide synthesis) or positive feedbacks at the network (protocell) level?

Theoretical models allow a quantitative analysis of what is possible, given the architecture of the system. We have previously shown that positive feedbacks involving FeS clusters chelated by amino acids can drive the growth of simple protocells composed of fatty acids and amino acids through CO_2_ fixation [50]. Experimental work confirmed two key predictions: robust protocells do assemble under the conditions proposed [51,52], and ‘biological’ FeS clusters form spontaneously in the presence of monomeric amino acids such as cysteine [53]. Here, we develop the protocell model to consider the synthesis of nucleotides via a branching protometabolism based on universally conserved pathways [54–57]. The model addresses how alterations in protometabolic flux affect protocell growth. Catalysis lowers the kinetic barriers to specific reactions, diverting flux down particular pathways [58]. But increased flux down one pathway (e.g. towards fatty acids) diminishes flux down other pathways (e.g. towards amino acids). We consider the full network of interactions to evaluate how nucleotide catalysis could contribute to protocell growth. These include nucleotide catalysis of CO_2_ fixation and specific protometabolic pathways [34,40,59–61] as well as nucleotide autocatalysis, and the cascade of feedbacks that ensue. The model reveals the necessary architecture of nucleotide catalysis that favours growth and examines whether such processes could generate enough nucleotides to facilitate polymerization and the emergence of genetic heredity.

## 2. Model overview

**Figure 1.**
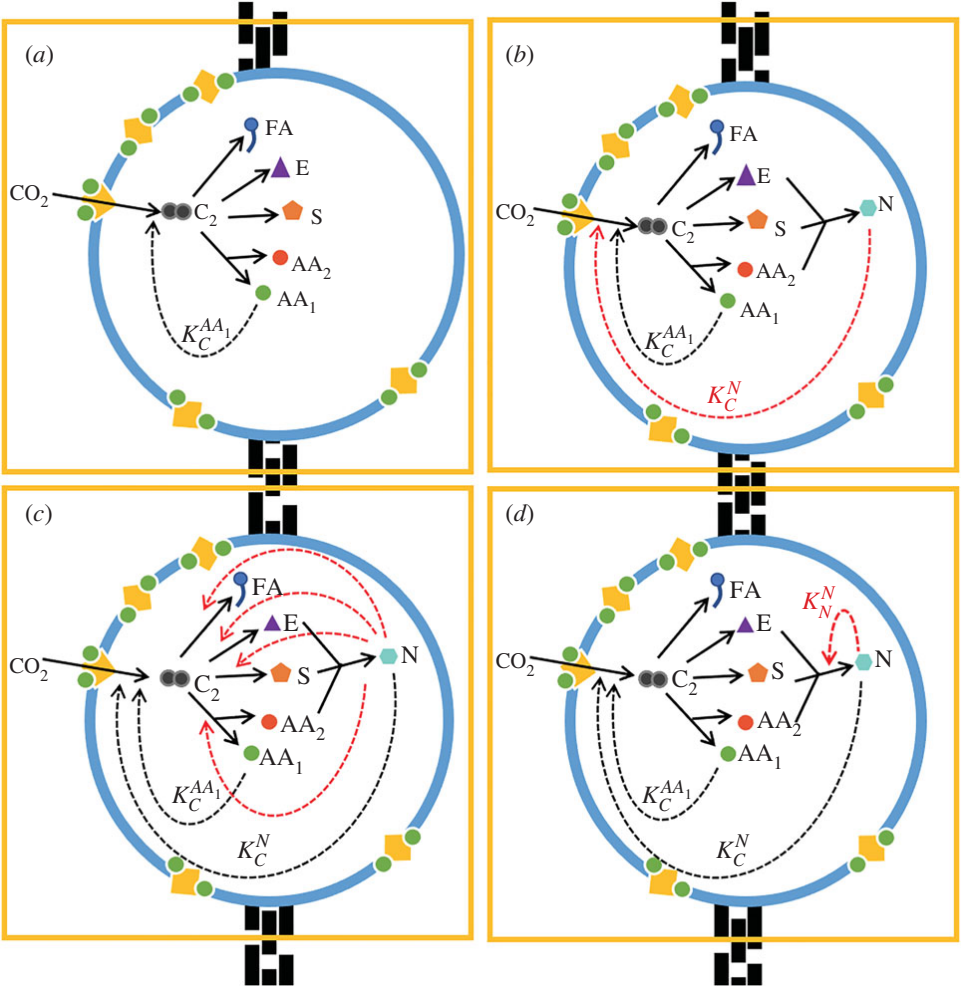
Models of positive feedbacks in protocells. Each panel depicts protocell models of autotrophic CO_2_ fixation driven by H_2_ (not shown) and catalysed by membrane-bound iron-sulfur clusters chelated by amino acids (yellow squares with green circles) under hydrothermal-type conditions. Fixed CO_2_ initially forms a simple two-carbon activated acetate, equivalent to acetyl CoA (*C*_2_), which acts as the primary substrate for a branching protometabolism based on the universally conserved core of biochemistry. (*a*) The null model considers the base-case in the absence of nucleotide formation in which the products are fatty acids (*FA*), amino acids, half of which (*AA*_1_) feedback on CO_2_ fixation, sugars (*S*) and energy (*E*). The subsequent models consider the catalytic function of nucleotides (*N*) produced from sugars (*S*), energy (*E*) and the other half of amino acids (*AA*_2_). In (*b*), nucleotides catalyse CO_2_ fixation ($K_{C}^{N}$) alone; in (*c*), nucleotides catalyse individual synthetic pathways ($K_{i}^{N}$, where *i* ∈ *FA*, *AA*_1_, *AA*_2_, *S*, *E*) in addition to CO_2_ fixation and in (*d*), nucleotides perform autocatalysis ($K_{N}^{N}$) in addition to CO_2_ fixation. Solid arrows represent synthesis reactions and dotted arrows represent catalysis and are shown in red for the variant models. Note that the energy currency *E* (equivalent to acetyl phosphate) is consumed by nucleotide synthesis, hence is equivalent to a substrate and is shown by a solid line.

We develop an earlier model of protocell metabolism grounded in prebiotic chemistry as the basis for our approach here [50]. The model assumes that the ions Fe^3+^ and S^2–^ are chelated by monomeric amino acids to form FeS clusters within protocells. These clusters associate with the membrane, where they draw on geochemically sustained proton gradients to facilitate CO_2_ fixation [40] (figure 1*a*). The first steps of CO_2_ fixation form a two-carbon prebiotic equivalent to acetyl CoA (e.g. methylthioacetate [20,34,60]) here labelled *C*_2_. We assume that Fe^3+^ and S^2–^ ions are not rate-limiting, so the rate of CO_2_ fixation is proportional to the number of amino acids capable of forming FeS clusters present in the cell.

The model is developed by introducing several metabolic branch points that result in *C*_2_ being converted into amino acids (*AA*), fatty acids (*FA*), sugars (*S*) and a primitive energy currency (*E*) (e.g. acetyl phosphate [60]; figure 1*b*). We do not model individual steps but assume that whole pathways are thermodynamically and kinetically favoured [62]. Other organic molecules would be produced from *C*_2_, but these are not considered further as they are irrelevant to turnover. The production of two types of amino acids is considered. The first set, *AA*_1_, chelates FeS clusters, which partition to the membrane and catalyse CO_2_ fixation. The second set, *AA*_2_, combines with sugars and energy currency to produce nucleotides (figure 1*b*). We arbitrarily assigned half of all amino acids to each set. The proportion of *C*_2_ channelled into the synthesis of each species is inferred from thermodynamic data under alkaline hydrothermal conditions ([13], table 1), which favours the production of amino acids and fatty acids (56.5% and 37.5%, respectively) over sugars (5%). No thermodynamic estimates are available for the production of the energy currency so the proportion of *C*_2_ channelled into it is set at a minimal level (1%) corresponding to low availability of inorganic phosphate [63–67]. We consider nucleotides can be catalytic, either by speeding up carbon fixation and thus indirectly increasing the production of all species in the protocell, or by increasing the production of individual species (including its own). The terms ‘weak’, ‘moderate’ and ‘strong’ catalysis are essentially arbitrary, chosen to clarify the behaviour of the system, and correspond to the specific catalytic rate constants specified in the figure legends. As the amount of fixed carbon available is finite, increasing the production of one species decreases the production of others. The model also describes protocell division, which occurs when the cell surface area (proportional to the fatty acid content) reaches a critical size. The daughter protocell inherits half the number of molecules of all species in the protocell.

**Table 1.** Main parameters used for simulations.

parameter	symbol	value	unit
rate constant for catalysis of CO_2_ fixation by nucleotides	$K_{C}^{N}$	10^3^	mol^−1^ dm^3^
rate constant for catalysis of nucleotide production by E	$K_{N}^{E}$	2	mol^−3^ dm^9^ s^−1^
rate constant for catalysis of nucleotide production by nucleotide cofactors	$K_{N}^{N}$	2	mol^−1^ dm^3^
saturating constant for catalysis of nucleotide production by nucleotides	$K_{i}^{N}$	1	cm^−1^ s^−1^
proportion of C_2_ molecules used to produce fatty acids	$\lambda_{FA}^{C}$	0.376	unitless
proportion of C_2_ molecules used to produce amino acids	$\lambda_{AA}^{C}$	0.564 (0.282 into each type of amino acid)	unitless
proportion of C_2_ molecules used to produce the energy currency (E)	$\lambda_{E}^{C}$	0.01	unitless
proportion of C_2_ molecules used to produce sugars	$\lambda_{S}^{C}$	0.05	unitless

The dynamics of protocell metabolism are modelled by a system of ordinary differential equations describing the change in the number of molecules of each species over time. These changes are determined by the number of stoichiometric reagents and catalysts present in the system. Besides amino acid catalysis of CO_2_ fixation, which is a feature of the null model (figure 1*a*), three additional catalytic positive feedbacks are considered: (i) catalysis of CO_2_ fixation by nucleotides (figure 1*b*), (ii) catalysis of individual protometabolic pathways alongside CO_2_ fixation (figure 1*c*) and (iii) nucleotide autocatalysis alongside CO_2_ fixation (figure 1*d*). A full description of the model is given in the electronic supplementary material.

## 3. Results

### (a) Nucleotide catalysis of CO_2_ fixation

**Figure 2.**
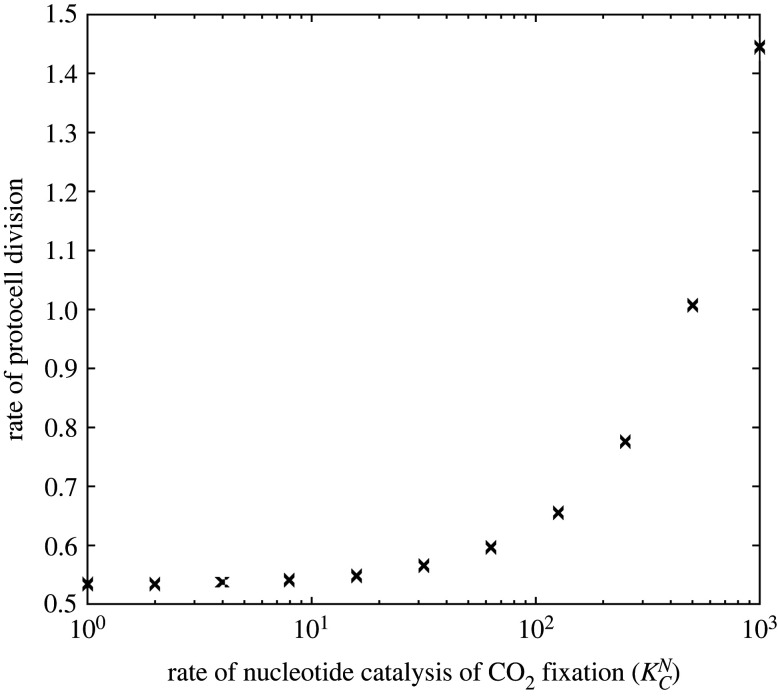
The effect of nucleotide catalysis of CO_2_ fixation ($K_{C}^{N}$) on the rate of cell division. Other parameter values are given in table 1.

The model assumes that CO_2_ fixation produces fatty acids (*FA*), which directly contribute to membrane growth, and amino acids, some of which chelate FeS clusters (*AA*_1_), providing a positive feedback loop that enhances CO_2_ fixation (figure 1*a*). Additional branch points generate further species (*AA*_2_, *S* and *E*), which contribute to nucleotide production (figure 1*b*). In the absence of nucleotide production (figure 1*a*) or nucleotide catalysis (i.e. $K_{C}^{N}=0$, figure 1*b*), this has no effect on cell growth. However, the additional metabolic branch points can be beneficial when nucleotides catalyse CO_2_ fixation (i.e. $K_{C}^{N}>0$; figure 1*b*). Nucleotide catalysis of CO_2_ fixation ($K_{C}^{N}$) generates a positive feedback, increasing the rate of cell division as the strength of catalysis increases (figure 2).

We found that the stable-state division rate and concentrations of all species are independent of initial concentrations when other parameters are kept constant. We include two examples of this in the electronic supplementary material (figures SI and S2): for increasing concentrations of all species in the cell (done by decreasing the number of fatty acids, as a proxy for cell size, and keeping the number of all other molecule types the same) (electronic supplementary material, figure S1), and for changes in the initial concentration of amino acids and thus the initial ratio of concentrations in the cell (electronic supplementary material, figure S2). In both analyses, cells with lower concentrations (of everything, or just amino acids) take longer to reach steady state, but the equilibrium concentrations are the same. We also repeatedly perturbed the concentrations of each species in the cell by adding stochastic segregation of molecules at cell division in a Moran model (see electronic supplementary material for details). At increasing levels of stochasticity (electronic supplementary material, figure S3a), the stable-state division rate remained unchanged (electronic supplementary material, figure S3b) as did the equilibrium concentrations of all species (illustrated by nucleotide concentrations, electronic supplementary material, figure S3c and S3d). Our analysis showed no bifurcation points or other unexpected dynamical features.

### (b) Nucleotide catalysis of individual pathways

**Figure 3.**
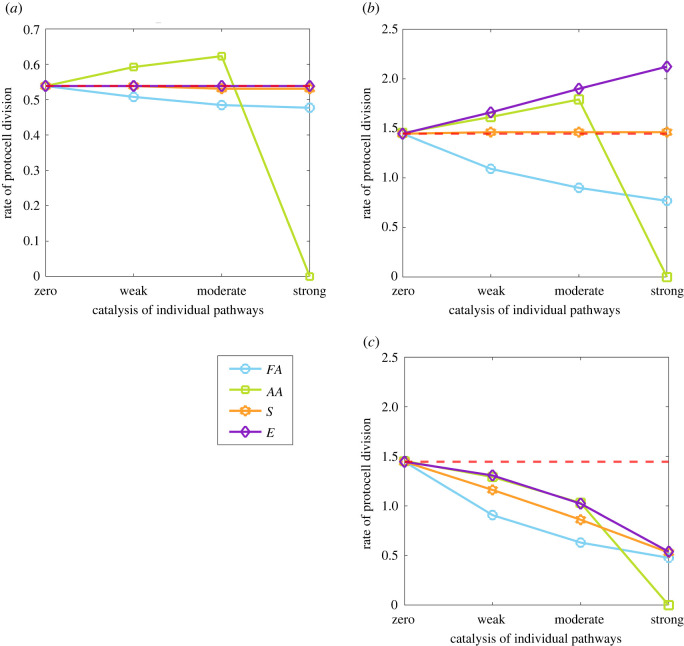
Impact of nucleotide catalysis of individual pathways on protocell division rate. Changes in protocell division per day are shown for nucleotide catalysis of the synthesis pathways of amino acids (*AA*, green), fatty acids (*FA*, blue), energy (*E*, purple) and sugars (*S*, orange). In each panel, nucleotide catalysis ranges from zero ($K_{i}^{N}=0$) to weak ($K_{i}^{N}=\;0.33$), moderate ($K_{i}^{N}=\;0.66$) and strong ($K_{i}^{N}=\;0.99$) of specific pathways *i* = *AA*, *FA*, *S*, *E*. These rates are considered in relation to (*a*) slow ($K_{C}^{N}=\;10^{0.9}$) and (*b*) fast ($K_{C}^{N}=\;10^{4}$) rates of CO_2_ fixation, given no cost to catalysis (*α* = 0). In (*c*), a cost to catalysis (*α* = 1) is introduced in proportion to the strength of nucleotide catalysis $K_{i}^{N}$ (with fast rates of CO_2_ fixation). The red dotted line indicates the number of protocell divisions per day with nucleotide catalysis of CO_2_ fixation alone (i.e. no catalysis of other synthesis pathways, as in figure 1*b*). Other parameter values are given in table 1.

**Figure 4.**
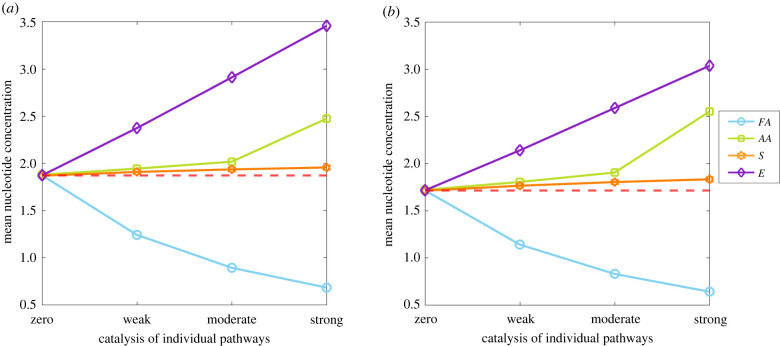
Nucleotide accumulation depending on catalysis of individual pathways. Changes in mean nucleotide concentration at equilibrium with increasing rates of nucleotide catalysis of specific pathways, using the same values as figure 3. (*a*) Slow ($K_{C}^{N}=\;10^{0.9}$) or (*b*) fast ($K_{C}^{N}=\;10^{4})\;$rates of CO_2_ fixation, assuming no cost to catalysis (*α* = 0). The red dotted line indicates the number of protocell divisions per day with nucleotide catalysis of CO_2_ fixation alone (i.e. no catalysis of other synthesis pathways). Other parameter values are given in table 1.

Taking catalysis of CO_2_ fixation as the base-case for comparison (figure 1*b*), we consider the consequences of nucleotide catalysis of individual branch points, enhancing the production of particular species in protocells (figure 1*c*). These consequences are observed through changes to the rate of protocell division (figure 3) and nucleotide concentration within protocells (figure 4).

Nucleotide catalysis of amino acid synthesis ($K_{AA}^{N}>0$) leads to a small increase in the rate of protocell division when the strength of $K_{AA}^{N}$ is weak or moderate (figure 3*a*). This is because increased amino acid synthesis (*AA*_1_) forms more FeS clusters, which promote CO_2_ fixation and so protocell growth through the positive feedback loop on amino acid synthesis. This positive feedback loop also generates more of the second set of amino acids (*AA*_2_), which contribute to nucleotide production, potentially enhancing the catalytic feedback of nucleotides on CO_2_ fixation. But that effect is self-limiting, as it does not overcome the disadvantage of channelling carbon away from other species. This disadvantage is clearly seen when catalysis increases the rate of the production of only one type of amino acid, leaving the other type unchanged (electronic supplementary material, figure S4). For these reasons, protocell growth and division collapses with strong catalysis of amino acids (figure 3*a*). If fixed carbon is excessively channelled into amino acid synthesis (when $K_{AA}^{N}\;$ strength is strong), that precludes fatty acid synthesis and inhibits growth and division. These results are independent of the strength of nucleotide catalysis on CO_2_ fixation. With fast nucleotide catalysis of CO_2_ fixation, the rate of protocell division increases, but the same collapse occurs with strong catalysis of amino acid synthesis for the same reasons—flux is diverted towards amino acids and away from fatty acids, which are necessary for protocell growth (figure 3*b*).

By contrast, if nucleotides catalyse fatty acid synthesis ($K_{FA}^{N}>0$), this invariably undermines protocell division (figure 3*a,b*). Even though fatty acids directly contribute to protocell growth (by increasing membrane surface area), there is no positive feedback loop associated with fatty acid synthesis. As a result, the advantage to protocell growth of channelling *C*_2_ into the production of fatty acids does not overcome the disadvantage of channelling *C*_2_ away from other products. Again, these results are independent of the catalytic strength of nucleotides on CO_2_ fixation, but are more dramatic with higher $K_{C}^{N}$ as this diverts a greater proportion of flux towards fatty acid synthesis and away from other useful products (figure 3*b*).

Catalysis of the pathways that synthesize an energy currency ($K_{E}^{N}>0$) or sugars ($K_{S}^{N}>0$) has a neutral or mildly deleterious effect on protocell growth when nucleotides catalyse CO_2_ fixation only weakly (figure 4*a*). The proportion of *C*_2_ used in the production of energy and sugars is too low to channel much fixed carbon away from the production of amino acids and fatty acids, even with strong catalysis of these pathways. But if nucleotide catalysis strongly favours CO_2_ fixation, there is a benefit to channelling more resources towards an energy currency, which becomes more apparent as $K_{E}^{N}$ increases (figure 4*b*). This difference reflects the assumption that less fixed carbon is channelled towards an energy currency than to sugar synthesis ($\lambda_{E}^{C}<\;\lambda_{S}^{C}$, table 1). Thus, making more energy currency helps because this species limits the production of nucleotides. Sugars are not limiting, so increasing their synthesis does not substantially influence nucleotide production. This interpretation is corroborated if we assume that more carbon is needed to make sugars (and so fewer sugar molecules are made per unit of *C*_2_) (electronic supplementary material, figure S5). In this case, the availability of sugars begins to limit nucleotide synthesis, so strong catalysis of sugar production now increases protocell growth.

### (c) Concentration of nucleotides

As well as altering the rate of protocell division (figure 3), nucleotide catalysis of individual pathways can alter the concentration of nucleotides within the protocell (figure 4). Nucleotide concentration only increases markedly when nucleotides catalyse the production of an energy currency ($K_{E}^{N}>0$). Again, this arises because energy is the limiting factor for nucleotide synthesis, so increasing the synthesis of an energy currency feeds through to a linear increase in nucleotide concentration within the protocell (figure 4*a*). That contrasts with catalysis of sugar production ($K_{S}^{N}>0$), which has, at best, a slightly positive effect on nucleotide concentration (figure 4*a*). The difference reflects the thermodynamic assumption that sugars are formed in excess of the requirements for nucleotide synthesis, so increasing their production does not substantially influence the concentration of nucleotides in the protocell.

Nucleotide catalysis of amino acid synthesis ($K_{AA}^{N}>0$) likewise has a marginal effect on nucleotide concentration, as channelling *C*_2_ towards amino acids retards the synthesis of sugars and an energy currency, whose production is essential to nucleotide generation (figure 4*a*). In addition, because weak or moderate amino acid catalysis of CO_2_ fixation drives protocell growth and division (figure 3*a*), nucleotide concentration is halved more regularly with each cell division, lowering their steady-state concentration. By contrast, strong catalysis of amino acid synthesis favours higher concentrations of nucleotides (figure 4*a*) because this collapses protocell growth (figure 3), allowing nucleotides to accumulate, while also synthesizing the amino acids needed for nucleotide synthesis (*AA*_2_) (figure 3*a*). This latter point explains the difference with fatty acid synthesis. Nucleotide catalysis of fatty acid synthesis ($K_{FA}^{N}>0$) not only reduces protocell division but also consistently lowers the concentration of nucleotides in protocells (figure 4*a*). That is because channelling *C*_2_ away from the precursors of nucleotide synthesis (amino acids, sugars and an energy currency) leads to a progressive decrease in the concentration of nucleotides.

These qualitative results are largely independent of the catalytic effect of nucleotides on CO_2_ fixation (figure 4). Faster nucleotide catalysis of CO_2_ fixation still generates similar levels of nucleotides in protocells (figure 4*b*). That is because fast catalysis of CO_2_ fixation ($K_{C}^{N}=\;10^{4}$) not only increases the rate of nucleotide production but also the rate of protocell growth and division, leaving nucleotide concentration at roughly the same level. A minor exception to this pattern is seen when nucleotides catalyse energy production, which leads to a slight decrease in the concentration of nucleotides with faster catalysis of CO_2_ fixation (i.e. Figure 4*b* versus figure 4*a*, purple lines). In this case, the energy currency drives nucleotides synthesis, which produces a stronger feedback on cell growth and division that in turn depletes nucleotide concentrations.

### (d) Costs of additional catalytic pathways

For the results described above, there is no cost to CO_2_ fixation through the introduction of additional catalysis of individual pathways (*α* = 0). This lack of cost is consistent with one type of nucleotide catalysing CO_2_ fixation and different types catalysing specific pathways. An alternative modelling possibility is that increased catalysis of specific pathways decreases the catalysis of CO_2_ fixation. This would be the case if the same type of nucleotide performed both catalytic functions (*α* > 0). In this case, catalysis of individual pathways leads to a decrease in the rate of CO_2_ fixation, which feeds through to a decrease in the rate of protocell division (figure 3*c*). This effect applies whichever metabolic branch point is catalysed: the stronger the competitive catalysis of specific pathways, the greater the negative effect on CO_2_ fixation and the corollary decline in protocell growth rate (figure 3*c*).

### (e) Nucleotide autocatalysis

**Figure 5.**
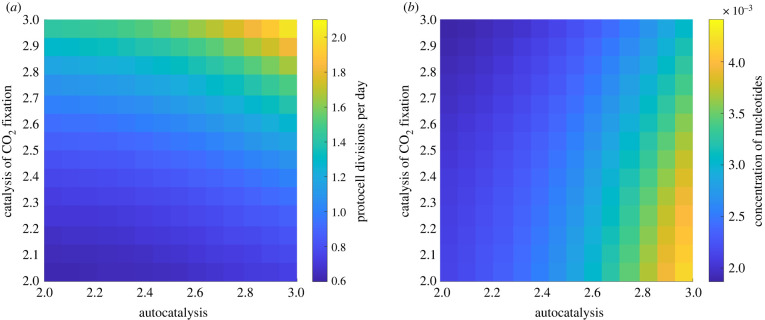
Impact of nucleotide autocatalysis and catalysis of CO_2_ fixation on protocell division rate and nucleotide concentration. Heat maps show (*a*) the number of protocell divisions per day and (*b*) the mean protocell concentration of nucleotides at equilibrium when varying the rate of nucleotide autocatalysis ($\log K_{N}^{N}$) and nucleotide catalysis of CO_2_ fixation ($\log K_{C}^{N}$). Other parameter values are given in table 1. (Online version in colour.)

A final possibility analysed is direct nucleotide catalysis of their own synthesis, i.e. direct rather than network autocatalysis (figure 1*d*). This can be advantageous for protocell growth when autocatalysis of nucleotides and catalysis of CO_2_ fixation are coupled and both $K_{N}^{N}$ and $K_{C}^{N}$ are high (figure 5*a*). However, this combination does not generate high concentrations of nucleotides in the protocell (figure 5*b*) for similar reasons to those noted above. Although nucleotides are produced at a faster rate, they do not accumulate because with a high rate of CO_2_ fixation their production is coupled to an increased rate of protocell growth and division. Nucleotide accumulation is greatest when autocatalysis ($K_{N}^{N}$) is high but catalysis of CO_2_ fixation ($K_{C}^{N}$) is low (figure 5*b*), which undermines the rate of protocell division (figure 5*a*) allowing nucleotide accumulation (figure 5*b*). A negative correlation between nucleotide autocatalysis ($K_{N}^{N}$) and catalysis of CO_2_ fixation ($K_{C}^{N}$) is observed if there is a cost interaction between the two types of catalytic pathways, meaning that the highest nucleotide concentrations are only generated at the cost of protocell division and *vice versa* (figure 5*a,b*).

## 4. Discussion

Prebiotic nucleotide synthesis is a necessary precursor for any form of RNA world. The model developed here explores how the catalytic activities of nucleotide monomers (naked cofactors) could contribute to the autotrophic growth of protocells and the concentration of nucleotides inside them. Assuming that the first protometabolic systems could, at best, generate trace amounts of nucleotides, nucleotide synthesis would need to be amplified through some form of positive feedback. We have analysed a range of different types of positive feedback, in which nucleotides catalyse CO_2_ fixation alongside a variety of branch points leading to the formation of particular molecular species (amino acids, fatty acids, sugars and a simple energy currency) as well as their own synthesis either in a direct (autocatalytic) or indirect manner (as a system outcome) [35,68,69]. The results show that positive feedbacks can drive growth but only under certain topological limits on catalysis, some of which can also lead to the amplification of nucleotide concentration.

We find that the most important way in which nucleotides can promote protocell growth is by steepening the driving force that pushes protometabolic flux through the entire network. This is primarily achieved by nucleotide catalysis of CO_2_ fixation, meaning the conversion of CO_2_ and H_2_ into the reactive precursors of protometabolism (assumed to be prebiotic thioesters [70,71]; figure 1). Nucleotide catalysis of individual protometabolic pathways can also enhance protocell growth, albeit with much tighter constraints (figure 1*c*). Catalysis of specific pathways does not directly change total flux through the system, but instead diverts flux down one pathway at the expense of others. This adjusts the ratio of products formed away from the thermodynamically assigned proportions. The only useful targets of downstream nucleotide catalysis are therefore amino acid synthesis (figure 3*a,b*) and energy currency, albeit in the latter case only when nucleotides strongly catalyse carbon fixation (figure 3*b*). Because both these processes promote CO_2_ fixation, either directly or indirectly, their catalysis ultimately enhances metabolic flux through the entire network.

However, there is a critical proviso when considering the catalysis of specific pathways. Strong catalysis of amino acid synthesis unbalances protometabolism, as it shifts too much carbon away from the fatty acids needed for the expansion of membrane surface area, collapsing protocell growth. This finding stresses the primacy of balanced flux for protocell growth: strong catalysis of specific pathways tends to unbalance protometabolism and collapse growth. Conversely, non-specific catalysis of general processes that apply across the network—promiscuous catalysis—limits this problem. In fact, most universally conserved nucleotide cofactors are promiscuous, including NADH, FADH, acetyl CoA and ATP [54,55,57,72]. These promiscuous cofactors catalyse equivalent processes, such as the transfer of hydrogen, electrons, phosphate, acetyl or methyl groups (all fundamental features of metabolism), in many pathways across the entire metabolic network [3,73,74].

Because cofactors can catalyse these reactions without their enzymes [47–49], the idea of an early era of biology when nucleotide monomers were the main catalysts has long held appeal [44,45]. Analysis of conserved biochemical pathways shows that NADH is indeed central to the earliest metabolic networks [68]. Precisely because nucleotide cofactors are promiscuous, they do not excessively divert carbon flux down any particular branch. The pterins and folates derived from GTP are a rare example of non-promiscuous cofactors, as they specialize in C1 metabolism. But in fact these cofactors prove the rule, as they promote CO_2_ fixation, driving balanced flux through the whole network. Similar processes to those performed by nucleotide cofactors occur with FeS clusters, metal ions or minerals, giving a seamless transition from geochemistry to biochemistry [53]. This also mirrors previous modelling work showing that, unlike modern phospholipid membranes, rudimentary bilayers made from fatty acids can freely exchange ions, allowing hydrothermal flow to maintain disequilibria (such as pH gradients) without the need for sophisticated pumps [75,76]. For autotrophic growth, only gases such as H_2_, CO_2_, NH_3_ and HS^−^ are needed, along with metal ions and protons, all of which readily cross leaky fatty acid membranes [76]. The idea that simplicity precedes complexity is therefore not just pleasing but necessary, for only simplicity works.

Nucleotide catalysis of specific pathways can affect nucleotide concentration within protocells. Accelerated synthesis of nucleotide precursors (amino acids, sugars and energy currency) results in varying levels of nucleotide accumulation. Nucleotide concentration only increases sharply when the energy currency pathway is favoured (figure 4), as energy is limiting; favouring this pathway feeds forward into a greater nucleotide synthesis. But nucleotide accumulation is lower when nucleotide catalysis of CO_2_ fixation is fast (figure 4*b*), as the increased number of nucleotides feeds back on protocell growth, speeding cell division and so halving nucleotide concentration more frequently. This important relationship between catalyst concentration and growth rate is emphasized by nucleotide autocatalysis. The prime example of this is purine synthesis, which relies on ATP, meaning that ATP is needed to make ATP [77]. If nucleotides catalyse CO_2_ fixation weakly, then autocatalysis contributes little to protocell growth, whatever the strength of autocatalysis (figure 5*a*,$\;K_{C}^{N}$ low, $K_{N}^{N}$ high). But with faster rates of CO_2_ fixation, cell division is strongly enhanced by autocatalysis (figure 5*a*, $K_{N}^{N}$ high, $\;K_{C}^{N}$ high). We anticipated that autocatalysis would generate excess nucleotides, but this is not the case in rapidly growing protocells (figure 5*b*). Instead, higher concentrations of nucleotides speed up protocell growth, lowering the nucleotide content as protocell division occurs more rapidly (albeit nucleotide synthesis is robust; electronic supplementary material, figure S3). This is an important general point: nucleotides will not accumulate to high concentrations in protocells if they have catalytic functions that speed up protocell growth and division. Thus, the premise for our model—protometabolic nucleotide synthesis requires catalytic positive feedbacks—precludes their continuous accumulation in actively growing protocells.

We have deliberately adopted a simple algorithm for cell divisions, which only takes into account the total number of fatty acids (used as a proxy for protocell surface area and volume). More sophisticated models of protocell division dynamics take into account factors such as osmotic pressure [78] and thermodynamic constraints [79]. These additional factors might limit the range of network topologies that give rise to viable protocells even further. However, because the objective of our simple model was to capture the general features of metabolic heredity before the emergence of genes, we have avoided complications arising from complex protocell division dynamics, addressed both experimentally and theoretically in the wider literature [80–83].

Our focus on autotrophic metabolic heredity also sets our work apart from the rich literature of protocell models dealing with the origin of heredity, much of which considers protocells in the context of an RNA world. This literature inspired our theoretical framework, notably Szathmary's stochastic corrector model [2], for its approach of heredity and multi-level selection of molecules in protocells, and Gánti's chemoton model [84], for its exploration of autocatalytic cycles in the context of protocells. But these and many other interesting protocell models [11,85–88] focus on the dynamics of RNA replicators or coded replicases, and so explore a later stage in evolution, after the advent of polymerization and genetic heredity [12,89]. While some have considered how metabolic networks can support protocell growth and division [90], the topologies explored there are theoretical and include importing ‘food’ molecules from the environment. These scenarios contrast with the autotrophic protocell model explored here, where the topology is constrained strictly by the core of autotrophic metabolism.

Is the assumption of autotrophic protometabolism valid? The relative values of the reaction rate constants used here, as well as specific concentrations and other parameters, allow us to capture the topological network constraints on growth, but omit many details. The reactions and catalytic steps assumed in the model link directly with phylogenetic reconstructions of early cells as obligate chemiosmotic autotrophs that grew from the reduction of CO_2_ by H_2_ [72]. While the rate of protocell growth in our model does vary with physical parameters such as CO_2_ concentration (electronic supplementary material, figure S6), the dynamics are not strongly influenced by such factors. The baseline proportion of carbon channelled into each metabolic branch is likewise based on thermodynamic estimates [13,15], and even highly stochastic inheritance at cell division does not substantially alter growth rates, nor the potential accumulation of nucleotides (electronic supplementary material, figure S3). The model is quite robust to the relative lowering of kinetic barriers through catalysis, but strong catalysis of particular pathways at the expense of others can collapse growth completely (figure 3) as this jeopardises the overall balance of protometabolism. Intriguingly, the thermodynamic estimates of products assigned to amino acids and fatty acids from CO_2_ fixation [13,15] appear to be fairly close to the optima for protocell growth (which is to say, serious perturbations from this balance collapse growth).

Experimental evidence for a protometabolism deriving from H_2_ and CO_2_ has garnered strength in recent years. Reactive equivalents to the C2 precursor modelled here have been synthesized experimentally under relevant prebiotic conditions [20,21]. Likewise, fatty acids and amino acids are not only thermodynamically favoured [13,15] but have been synthesized experimentally under relevant conditions [22,27–31]. While sugars, notably ribose, have also been synthesized under similar conditions [32,33], thermodynamically these are less favoured [13,15], so in the model we assumed they formed at arbitrarily low levels (5% of C2, table 1). We also assumed that acetyl phosphate (a plausible prebiotic energy currency [34,59–61,91,92]) is formed at low levels, as the prebiotic availability of phosphate was probably limiting [63–67], and experiments achieved only low yields of acetyl phosphate under relevant conditions [60].

While there is no definitive evidence that this full set of protometabolic reactions can take place in a single setting, such as protocells, most experimental investigations share commonalities. In particular, in all the examples mentioned above, the chemistry takes place in an aqueous system. pH can vary, with some syntheses taking place in mildly acidic (approx. 4–5) [22,23,61] or neutral pH [93], and many other syntheses in alkaline (approx. 8–10) conditions [19,20,33,43,94]. Such a pH range is entirely consistent with an alkaline hydrothermal vent hypothesis, and we would anticipate that protocells would be exposed to much of this range. Indeed, we argue that pH gradients are needed to drive flux through protometabolic networks [40,41,95]. The catalysts used are typically period 4 transition metal ions, most prominently iron and copper [16–19,22,23,25,43,61,93] or their minerals e.g. FeS, Fe_x_(OH)_y_ and Ni_3_Fe [19,27,94]. Period 4 metal ions are perhaps uncommon, but certainly not rare, and would likely be present in most environmental systems [96,97]. Other synthetic reactions employ biologically relevant cofactors, e.g. pyridoxal-5-phosphate, but in the absence of enzymatic architecture [93], equivalent to the ‘naked cofactors’ modelled here. With respect to time scales, these syntheses vary from near instantaneous to days [17–20,22,23,27,43,33,61,93–95], which is in the right order to sustain an autotrophic protometabolism (as opposed to much longer time scale heterotrophic origins). Temperature requirements are variable, but mostly fall within the standard atmospheric range of 20–100°C [17–20,22,23,27,33,43,61,93–95], again common in alkaline hydrothermal systems. The exception is fatty acid synthesis [28] which required temperatures of approximately 175°C, but such temperatures are not unusual in higher pressure aqueous systems, or deeper in the crust [98]. Overall, the overlapping conditions in this patchwork of metabolic reactions broadly support our assumption of a coherent protometabolic network.

Assuming that such an autotrophic protometabolism is indeed feasible, the model developed here shows that there are constraints on how flux must operate in protocells. Catalysts must facilitate balanced flux through protometabolism to promote fast protocell growth via positive feedbacks. Selection for fast growth is likely to oppose the accumulation of catalytic nucleotides within protocells, albeit positive feedbacks favouring the synthesis of an energy currency can increase nucleotide concentrations somewhat. Most importantly, the model gives a new context for the emergence of the genetic code in autotrophic protocells [99]. Whatever processes allowed polymerization of nucleotides in this setting, the introduction of short, random RNA sequences inside replicating protocells offers an immediate informational context. Rather than ‘inventing’ information from nothing, the first peptides or ribozymes would drive protocell growth through interactions with cofactors, promoting balanced flux through the same branching network that is still conserved today. So protocell growth gives meaning to information from the origins of polymerization, laying the foundations for the emergence of genetic heredity.

## Data Availability

All primary modelling data for this paper are publicly accessible on the github digital repository: https://github.com/raquelnpalmeira/limits_of_metabolic_heredity. The data are provided in the electronic supplementary material [100].
